# Prevalence of urgent hospitalizations caused by adverse drug reactions: a cross-sectional study

**DOI:** 10.1038/s41598-024-56855-z

**Published:** 2024-03-13

**Authors:** Junpei Komagamine

**Affiliations:** 1 Department of Emergency and Critical Care Medicine, NHO Tokyo Medical Center, 2-5-1, Higashigaoka, Meguro, Tokyo Japan; 2 Department of Clinical Research, NHO Tochigi Medical Center, Utsunomiya, Japan

**Keywords:** Adverse drug reaction, Hospitalization, Polypharmacy, Medical research, Epidemiology, Epidemiology

## Abstract

Adverse drug reactions account for a substantial portion of emergency hospital admissions. However, in the last decade, few studies have been conducted to determine the prevalence of hospitalization due to adverse drug reactions. Therefore, this cross-sectional study was conducted to determine the proportion of adverse drug reactions leading to emergency hospital admission and to evaluate the risk factors for these reactions. A total of 5707 consecutive patients aged > 18 years who were emergently hospitalized due to acute medical illnesses between June 2018 and May 2021 were included. Causality assessment for adverse drug reactions was performed by using the World Health Organization-Uppsala Monitoring Centre criteria. The median patient age was 78 years (IQR 63–87), and the proportion of women was 47.9%. Among all the hospitalizations, 287 (5.0%; 95% confidence interval (CI) 4.5–5.6%) were caused by 368 adverse drug reactions. The risk factors independently associated with hospitalization due to adverse drug reactions were polypharmacy (OR 2.66), age ≥ 65 years (OR 2.00), and ambulance use (OR 1.41). Given that the population is rapidly aging worldwide, further efforts are needed to minimize hospitalizations caused by adverse drug reactions.

## Introduction

An adverse drug reaction (ADR) is defined as a harmful or uncomfortable event due to medication^1^. Older age and polypharmacy, which is defined as the use of more or five regular medications, are associated with an increased risk of ADRs^2,3^. Given the aging population worldwide, monitoring and minimizing the burden of ADRs are important.

The most frequent encounter for ADRs is ambulatory care^4^. According to ambulatory care-based studies^4,5^, approximately one in every eight outpatients have at least one ADR during no more than one year. However, monitoring ADRs in an ambulatory setting is laborious^5–7^. Therefore, monitoring emergency department (ED) visits or urgent hospitalizations due to ADRs is an alternative strategy^6^. Previous meta-analyses of observational studies^2,8–13^ reported that 5–10% of hospital admissions were caused by ADRs. Moreover, approximately half of the hospitalizations due to ADRs were preventable^11,12^.

However, in the last decade, few studies have been conducted to determine the prevalence of hospitalization due to ADRs^14–16^. Moreover, passive surveillance systems reporting ADRs underestimate their prevalence because of underreporting and lack of awareness by clinicians^17,18^. Given that a substantial proportion of hospitalizations is caused by ADRs, it is important to establish the accurate prevalence of hospitalization due to ADRs. Therefore, a cross-sectional study was conducted to determine the prevalence of hospitalization due to ADRs and associated risk factors.

## Results

The baseline characteristics of the 5707 patients hospitalized for acute medical illnesses are shown in Table 1. The median patient age was 78 years (interquartile range (IQR) 63–87), 2605 (45.6%) were women, 4123 (72.3%) were independent in activities of daily living (ADLs), and 3217 (56.4%) had used an ambulance. The median Charlson Comorbidity Index score was one (IQR 0–3), 1011 patients (17.7%) had chronic kidney disease, and 2743 (48.1%) took more or five regular medications at admission. The most common chief complaint was fever or chills (n = 1278, 22.4%), followed by dyspnea (n = 793, 13.9%), abdominal pain or discomfort (n = 394, 6.9%), and chest pain or discomfort (n = 351, 6.2%) (eTable 1 in the Supplemental file). The most common final clinical diagnoses leading to hospitalization were acute heart failure (n = 623, 10.9%), followed by COVID-19 (n = 533, 9.7%), pneumonia or pneumonitis (n = 524, 9.2%), gastrointestinal bleeding (n = 361, 6.3%), acute coronary syndrome (n = 259, 4.5%), and stroke or transient ischemic attack (n = 239, 4.2%) (eTable 2 in the Supplemental file).

**Table 1:** Characteristics of the 5707 patients hospitalized for acute medical illnesses.

Characteristics	Total (n = 5707)	Hospitalization due to ADRs^a^	
		Yes (n = 287)	No (n = 5420)
Age (year), median (IQR)	78 (63–87)	82 (74–88)	77 (62–86)
Aged 65 years or older, n (%)	4204 (73.7)	257 (89.6)	3947 (72.8)
Women, n (%)	2605 (45.6)	152 (53.0)	2453 (45.3)
Japanese	5625 (98.6)	287 (100.0)	5338 (98.5)
Activities of daily living			
Independent	4123 (72.4)	200 (70.2)	3923 (72.5)
Partially dependent	1306 (22.9)	74 (26.0)	1232 (22.8)
Dependent	267 (4.7)	11 (3.9)	256 (4.7)
Median Charlson Comorbidity Index score (IQR)	1 (0–3)	2 (1–3)	1 (0–3)
Medical history, n (%)			
Stroke	909 (15.9)	66 (23.0)	843 (15.6)
Myocardial infarction	366 (6.4)	27 (9.4)	339 (6.3)
Heart failure	791 (13.9)	61 (21.3)	730 (13.5)
Dementia	855 (15.0)	52 (18.1)	803 (14.8)
COPD or asthma	573 (10.0)	23 (8.0)	550 (10.2)
Chronic kidney disease	1011 (17.7)	73 (25.4)	938 (17.3)
Diabetes mellitus	1334 (23.4)	97 (33.8)	1237 (22.8)
Hypertension	3197 (56.0)	218 (76.0)	2979 (55.0)
Peptic ulcer	475 (8.3)	36 (12.5)	439 (8.1)
Number of regular medications			
Median (IQR)	4 (1–7)	7 (4–9)	4 (1–7)
Five or more medications, n (%)	2743 (48.1)	213 (74.2)	2530 (46.7)
Ten or more medications, n (%)	593 (10.4)	70 (24.4)	523 (9.7)
Ambulance use, n (%)	3217 (56.4)	190 (66.2)	3027 (55.9)
Median days of hospital stay (IQR)	10 (5–20)	10 (5–22)	10 (5–20)
In-hospital death, n (%)	445 (7.8)	15 (5.2)	430 (7.9)

*ADR* adverse drug reaction, *COPD* chronic obstructive pulmonary disease, *IQR* interquartile range.

A total of 494 medications contributed to adverse events at admission (Table 2 and eTable 3 in the Supplemental file). Of those, 368 medications (74.5%) were judged to cause 287 hospitalizations due to ADRs. The most common categories of medications leading to hospitalization were cardiovascular agents (n = 83, 22.6%), musculoskeletal agents (n = 62, 16.8%), antithrombic agents (n = 49, 13.3%), psychotropic agents (n = 39, 10.6%), and antidiabetic agents (n = 37, 10.1%). Medications in these five categories accounted for more than 70% of all ADRs leading to hospitalization.

**Table 2:** Most common categories of medications associated with adverse drug reactions at admission.

Categories^a,b^	Medications leading to hospitalization^c^ (n = 368)	Medications associated with any ADRs (n = 494)
Cardiovascular agents	83 (22.6)	117 (23.7)
Antiarrhythmic agents	14 (3.8)	16 (3.2)
Diuretics	30 (8.2)	51 (10.3)
Beta-blocking agents	11 (3.0)	14 (2.8)
RAS inhibitors	17 (4.6)	22 (4.5)
Musculoskeletal agents	62 (16.8)	67 (13.6)
NSAIDs	41 (11.1)	44 (8.9)
COX-2 inhibitors	21 (5.7)	22 (4.5)
Antithrombic agents	49 (13.3)	102 (20.6)
Antiplatelet agents	12 (3.3)	18 (3.6)
Anticoagulant agents	12 (3.3)	33 (6.7)
Psychotropic agents	39 (10.6)	48 (9.7)
Benzodiazepines	23 (6.3)	26 (5.3)
Antipsychotics	11 (3.0)	17 (3.4)
Antidiabetic agents	37 (10.1)	39 (7.9)
Sulfonylureas	15 (4.1)	15 (3.0)
Insulins	13 (3.5)	14 (2.8)
Psychoanaleptics	18 (4.9)	18 (3.6)
Antineoplastic agents	17 (4.6)	20 (4.0)
Antimicrobial agents	13 (3.5)	19 (3.8)
Herbal medications	12 (3.3)	17 (3.4)

^a^These included medications representing more than 3% of all medications.

^b^These categories of medications were based on the World Health Organization Anatomical Therapeutic Chemical classification.

^c^A total of 368 medications resulted in 287 hospitalizations.

*ADR* adverse drug reaction, *COX-2* cyclooxygenase-2, *DOAC* direct-acting oral anticoagulant, *DPP-4* dipeptidyl peptidase IV, *NSAIDs* nonsteroidal anti-inflammatory drugs, *RAS* renin-angiotensin system.

For the primary outcome, the proportion of patients who were hospitalized due to ADRs among all patients hospitalized for acute medical illnesses was 5.0% (95% CI 4.5–5.6%). According to our multivariable analysis, the risk factors independently associated with hospitalization due to adverse drug reactions were polypharmacy (odds ratio (OR) 2.66; 95% CI 2.00–3.54), age ≥ 65 years (OR 2.00; 95% CI 1.34–3.00), and ambulance use (OR 1.41; 95% CI 1.41–1.81) (Table 3). For the secondary outcome, the proportion of patients who had any ADRs at admission among all patients hospitalized for acute medical illnesses was 6.6% (95% CI 6.0–7.3%).

**Table 3:** Univariable and multivariable analyses of predictive factors for hospitalization caused by adverse drug reactions.

Variables	Hospitalization due to adverse drug reactions	
	Unadjusted OR (95% CI)	Adjusted OR^a^ (95% CI)
Aged 65 years or older	3.20 (2.18–4.69)*	2.00 (1.34–3.00)*
Women	1.36 (1.07–1.73)*	1.23 (0.96–1.57)
Ambulance use	1.55 (1.21–1.99)*	1.41 (1.09–1.81)*
CCI score^b^	1.12 (1.06–1.19)*	1.01 (0.94–1.08)
Chronic kidney disease	1.63 (1.24–2.15)*	1.17 (0.87–1.56)
Polypharmacy^c^	3.29 (2.51–4.31)*	2.66 (2.00–3.54)*

The threshold for statistical significance was set at *p* < 0.05. Asterisks indicate a significant association between selected variables and unplanned admission due to adverse drug reactions.

^a^The variables adjusted for were age, sex, ambulance use, CCI score, chronic kidney disease, and polypharmacy.

^b^Continuous variables were used.

^c^Polypharmacy was defined as five or more medications.

*CCI* Charlson Comorbidity Index, *CI* confidence interval, *OR* odds ratio.

The post hoc analysis showed that the prevalence rates of hospital admissions due to ADRs during the pre-COVID-19 pandemic and COVID-19 pandemic periods were 6.1% and 3.8%, respectively (eTable 4 in the Supplemental file). The prevalence of hospitalizations due to ADRs was significantly lower during the COVID-19 pandemic period than during the pre-COVID-19 pandemic period (*p* < 0.001).

## Discussion

The present study showed that approximately 5% of urgent hospital admissions were caused by ADRs. Our findings are consistent with those of past meta-analyses^10,13^ of observational studies using data before 2012 showing that 5–10% of hospital admissions were caused by ADRs. This means that the prevalence of hospital admissions due to ADRs has not significantly improved during the last decade. However, compared with the findings of a previous recent study^19^ using almost the same methods, the prevalence of hospital admissions due to ADRs in the present study was reduced by approximately half. In Japan, the “Proper Medication Guideline for Older Adults” was published for healthcare providers in May 2018^20^, and polypharmacy reduction incentives were initiated in 2016^21^ and 2018^22^. A recent study reported that the prevalence of polypharmacy among elderly individuals subsequently decreased in Japan^23^. Therefore, it is possible that the national strategy for polypharmacy among elderly patients in Japan might have reduced hospital admissions due to ADRs. However, according to post hoc analysis, the prevalence of hospital admissions due to ADRs significantly decreased from the pre-COVID-19 pandemic period to the COVID-19 pandemic period. During the COVID-19 pandemic, the number of hospital visits in Japan substantially decreased^24^. Therefore, a reduction in hospital visits and increase in hospital admissions due to COVID-19 might have resulted in a decrease in the prevalence of hospital admissions due to ADRs. Nonetheless, additional studies are needed to confirm this hypothesis and monitor the prevalence of hospital admissions induced by ADRs since the COVID-19 pandemic.

In the present study, cardiovascular agents, nonsteroidal anti-inflammatory drugs (NSAIDs), antithrombic agents, psychotropic agents, and antidiabetic agents accounted for more than two-thirds of the medications leading to hospitalization. This finding is similar to that of past studies showing that the most common categories of medications leading to hospitalization included cardiovascular agents, antithrombic agents, psychotropic agents, NSAIDs, and antidiabetic agents^3,4,6,12,13^. Although the proportion of opioid use was lower in the present study than in past studies^13,25^, this difference might be attributed to the lower consumption of opioids in Japan than in European countries and the United States^26^. Previous ^19,27^ and recent studies showed that the proportion of psychotropic agents in medications causing hospitalization was greater than that in other countries^3,4,6,12,13^. Most of the patients included in the present study were 65 years or older. Given that psychotropic agents for elderly patients are considered potentially inappropriate medications in most cases^28,29^, further efforts to minimize the harmful effects of psychotropic agents are needed by implementing proper medication guidelines^20,30^. Moreover, in the present study, polypharmacy and age ≥ 65 years were independent risk factors associated with hospitalization due to ADRs. Our findings are consistent with those of a previous systematic review^13^ regarding hospitalization due to ADRs. Therefore, deprescribing interventions for elderly patients with polypharmacy is also important.

In the present study, ambulance use was independently associated with an increased risk of hospitalization due to ADRs. This indicated that patients who were hospitalized due to ADRs had more severe symptoms that necessitated ambulance use than those who were not. To our knowledge, no studies have ever been conducted to investigate the association between ambulance use and hospitalization induced by ADRs^3^. Therefore, further studies are needed.

This study has several limitations. First, the present study was limited to patients hospitalized in the internal medicine ward of a single center. Second, a substantial proportion of patients were excluded due to insufficient information on documented medication history and medical history. Third, ADRs were screened based on information from electronic medical records documented in usual care. Therefore, some assessments of the causality of ADRs might have been inaccurate. Fourth, hindsight bias might have overestimated the prevalence of hospitalizations due to ADRs^31^. Fifth, ADRs were assessed by a single investigator. Therefore, the assessments of the causality of ADRs might have been inaccurate. Sixth, the preventability of ADRs was not assessed. Seventh, the inclusion of patients who were readmitted during the study period in the present study might overemphasize the characteristics of those patients. Finally, a medication list of trigger symptoms was not used to identify the medications that might have caused adverse events. Therefore, the prevalence of ADRs might have been underestimated.

In conclusion, one in every twenty emergency hospital admissions was caused by ADRs. Polypharmacy and old age were associated with an increased risk of hospitalization due to ADRs. Given the rapidly aging populations worldwide, efforts to mitigate hospitalizations due to ADRs are needed.

## Methods

### Study design and setting

A single-center retrospective cross-sectional study using chart reviews was conducted at NHO Tochigi Medical Center to determine the prevalence of hospitalization caused by ADRs among all emergency hospital admissions. NHO Tochigi Medical Center is a 350-bed general community hospital in Utsunomiya, Japan, and is one of the two largest acute care hospitals providing care for approximately 0.5 million people in this area. This research was approved and the requirement for individual informed consent was formally waived by the Medical Ethics Committee of NHO Tochigi Medical Center (No. 2019-2) because deidentified data were collected without contacting the patients. This research was conducted in accordance with the Ethical Guidelines for Epidemiological Research in Japan and the Declaration of Helsinki.

### Inclusion and exclusion criteria

All consecutive patients aged > 18 years who were urgently hospitalized in the internal medicine ward of NHO Tochigi Medical Center due to acute medical illnesses from June 2018 to May 2021 were included. If the same patient was admitted two or more times during the study period, the admissions were evaluated separately. Patients who were planned to be hospitalized for diagnostic procedures, education, treatment, or short-term care were excluded. In this study, data collected in usual care were used. Therefore, patients with insufficient information on regular medications or medical history at admission documented in the electronic medical records were also excluded. During the study period, 7468 patients were hospitalized. A total of 5707 patients who met the inclusion criteria were included in the final analysis (eFigure 1 in the Supplemental file).

### Data collection and screening

Information on age, sex, medical history, medications, primary diagnosis at hospitalization, and prognosis was collected as deidentified data from electronic medical records. For the primary diagnosis, the documented diagnosis in the discharge summary was used. For information on prescribed medications, a comprehensive medication list documented by pharmacists as usual care was used. The type of medication was classified according to the World Health Organization Anatomical Therapeutic Chemical classification. Based on the medication list and the patients’ medical histories, physical findings, and laboratory test results from the electronic medical records, the medications that could cause ADRs were screened. During the study period, all patients who were hospitalized in the internal medicine ward of the hospital were screened within a few days after admission as routine care. All medications that the patients took were evaluated for whether they caused ADRs at hospital admission. If needed, the principal physicians were contacted regarding the possibility of ADRs due to specific medications. The patients were subsequently followed up until discharge by using the electronic medical records.

### Outcome measures

The primary outcome was the proportion of patients who were hospitalized due to ADRs among hospitalized patients with acute medical illnesses. Based on a previous report^1^, ADRs were defined as harmful or unpleasant reactions resulting from an intervention related to the use of a medicinal product, which predicts risks from future administration and warrants prevention or specific treatment, alteration of the dosage regimen, or withdrawal of the product. Unpleasant reactions included any new symptoms or signs that were presumed to be caused by the medications. However, ADRs associated with treatment failure or withdrawal of medications were not included. Intentional drug abuse or unintentional medication overdose were not included as ADRs. The secondary outcome was the proportion of patients who had any ADRs at admission among hospitalized patients with acute medical illnesses.

The causality of ADRs was assessed according to the World Health Organization-Uppsala Monitoring Centre (WHO-UMC) criteria^32^. The medication was judged to cause adverse events if both of the following criteria were met: (1) there was a “certain” or “probable” causal association between the medication and adverse events based on the WHO-UMC criteria^32^; and (2) the medication was stopped or its dose was reduced by the principal physician caring for the patient during the index hospitalization. The contribution of the ADR to hospitalization was evaluated based on the Hallas criteria^33^. The patients for whom problems due to ADRs were “dominant” leading to hospitalization were judged to be hospitalized due to ADRs. These assessments were performed by a single investigator (J.K.).

### Statistical analysis

Initially, approximately 10,000 patients were planned to be included. This sample size would provide a precision of 0.5% for calculation of the 95% confidence interval (CI) of the primary outcome, assuming that the proportion of patients who were hospitalized due to ADRs was 7%, which was based on previous Japanese studies^19,27^ and systematic reviews^10,13^. However, the recent coronavirus disease 2019 (COVID-19) pandemic made it difficult to continue enrollment due to a shortage of healthcare providers. Therefore, the sample size of this study was reduced.

Descriptive statistics are used to report the baseline characteristics of the study population. For the primary outcome, the proportion of patients who were hospitalized due to ADRs among all the hospitalized patients was calculated. For the secondary outcome, the proportion of hospitalized patients who had any ADRs at admission among all hospitalized patients was calculated. The 95% CIs were calculated for these outcomes. Multivariate analysis using binary logistic regression was performed to determine the predictive factors associated with ADRs leading to hospitalization. We examined the associations between the primary outcome and age, sex, ambulance use, Charlson Comorbidity Index score, chronic kidney disease, and polypharmacy at admission. Polypharmacy was defined as the use of five or more medications^34^. The clinical features of patients who are transferred or not transferred by ambulance to the emergency department are different^35^. Therefore, ambulance use was chosen as one of the variables. The COVID-19 pandemic began during the study period. In the region of the hospital, the first hospital admissions of COVID-19 patients occurred in February 2020. Therefore, in a post hoc analysis, the proportion of patients who were hospitalized due to ADRs between the pre-COVID-19 pandemic period (June 2018 to January 2020) and the COVID-19 pandemic period (February 2020 to May 2021) was compared by using Fisher’s exact test. Excel statistical software version 4.04 (Bellcurve for Excel; Social Survey Research Information Co., Ltd., Tokyo, Japan) and Stata version 18.0 (StataCorp, USA) were used for these analyses. A *p* value < 0.05 indicated statistical significance.

### Supplementary Information

Supplementary Information.

## Data Availability

All data relevant to the study are included in the article or uploaded as supplementary information.
