# Supplementary material for: Prevalence of urgent hospitalizations caused by adverse drug reactions: a cross-sectional study
Source: Sci Rep. 2024 Mar 13;14:6058. doi: 10.1038/s41598-024-56855-z (PMC10937656; doi:10.1038/s41598-024-56855-z)
Supplement: Supplementary file 1 — Supplementary Information. [file 41598_2024_56855_MOESM1_ESM.docx]

A total of 7468 hospitalizations

Excluded due to nonurgent admissions (n = 1345)

- Elective admission for diagnostic or interventional procedures (n = 1121)
- Education for diabetes mellitus (n = 35)
- Short-term care (n = 22)
- Others (n = 167)

Hospitalizations due to acute medical illnesses (n = 6123)

Excluded (n = 498)

- Insufficient data on medical history and medications (n = 468)
- Younger than 18 years (n = 30)

Hospitalizations included in the final analysis (n = 5707)

**eFigure 1.** Flow chart of the 5707 patients included in this study.

**eTable 1.** Chief complaints of the 5707 patients hospitalized for acute medical illnesses.

| **Chief complaints, n (%)** | **Total**  **(n = 5707)** | **Hospitalization due to ADRs^a^** | |
| --- | --- | --- | --- |
|  |  | **Yes (n = 287)** | **No (n = 5420)** |
| Fever or chills  Dyspnea  Abdominal pain or discomfort  Chest pain or discomfort  Altered consciousness  Hematemesis, melena, and bloody stool  Weakness  Dizziness or vertigo  Nausea or vomiting  Anorexia  Cough or sputum  Syncope or transient loss of consciousness  Malaise  Hemiparesis | 1278 (22.4)  793 (13.9)  394 (6.9)  351 (6.2)  326 (5.7)  325 (5.7)  287 (5.0)  243 (4.3)  238 (4.2)  200 (3.5)  146 (2.6)  88 (1.0)  80 (1.4)  63 (1.1) | 9 (3.1)  59 (20.6)  7 (2.4)  2 (0.7)  42 (14.6)  32 (11.2)  18 (6.3)  8 (2.8)  26 (9.1)  22 (7.7)  0 (0.0)  12 (4.2)  6 (2.1)  1 (0.4) | 1269 (23.4)  734 (13.5)  387 (7.1)  349 (6.4)  284 (5.2)  293 (5.4)  269 (5.0)  235 (4.3)  212 (3.9)  178 (3.3)  146 (2.7)  76 (1.4)  74 (1.4)  62 (1.1) |

ADR, adverse drug reaction.

**eTable 2.** Distributions of primary diagnoses for the included 5707 patients.

| **Primary diagnosis for hospitalization^a^, n (%)** | **Total**  **(n = 5707)** | **Hospitalization due to ADRs^a^** | |
| --- | --- | --- | --- |
|  |  | **Yes (n = 287)** | **No (n = 5420)** |
| Acute heart failure  COVID-19  Pneumonia or pneumonitis^b^  Gastrointestinal bleeding  Acute coronary syndrome  Stroke or transient ischemic attack  Urinary tract infection  Biliary tract infection  Malignancy  Vestibular disease  Medication-related adverse events^c^  Fever (unspecified)  Soft tissue infection  Bowel obstruction  Asthma or COPD exacerbation  Arrhythmia  Epilepsy  Rheumatic disease  Pancreatitis  Liver cirrhosis | 623 (10.9)  553 (9.7)  524 (9.2)  361 (6.3)  259 (4.5)  239 (4.2)  226 (4.0)  191 (3.4)  163 (2.9)  154 (2.7)  126 (2.2)  111 (1.9)  100 (1.8)  87 (1.5)  83 (1.5)  72 (1.3)  66 (1.2)  62 (1.1)  61 (1.1)  59 (1.0) | 52 (18.3)  0 (0.0)  4 (1.4)  41 (14.3)  0 (0.0)  3 (1.1)  0 (0.0)  0 (0.0)  0 (0.0)  0 (0.0)  125 (43.6)  0 (0.0)  0 (0.0)  1 (0.4)  1 (0.4)  7 (2.4)  0 (0.0)  0 (0.0)  0 (0.0)  0 (0.0) | 571 (10.5)  553 (10.2)  520 (9.6)  320 (5.9)  259 (4.8)  236 (4.4)  226 (4.2)  191 (3.5)  163 (3.0)  154 (2.8)  1 (0.0)  111 (2.1)  100 (1.9)  86 (1.6)  82 (1.5)  65 (1.2)  66 (1.2)  62 (1.1)  61 (1.1)  59 (1.1) |

^a^These included diseases representing more than 1% of all hospitalizations.

^b^This did not include COVID-19.

^c^This was not an investigator-defined adverse drug reaction but rather based on clinical documentation by the principal physicians in usual care.

ADR, adverse drug reaction; COPD, chronic obstructive pulmonary disease; COVID-19, coronavirus disease 2019.

**eTable 3.** Most common categories of medications associated with adverse drug reactions at admission.

| **Categories^a^** | **Medications leading to hospitalization^b^ (n = 368)** | **Medications associated with any ADR (n = 494)** |
| --- | --- | --- |
| **Cardiovascular agents**  Antiarrhythmic agents  Diuretics  Beta-blocking agents  Calcium channel blockers  RAS inhibitors  Lipid-modifying agents | 83 (22.6)  14 (3.8)  30 (8.2)  11 (3.0)  10 (2.7)  17 (4.6)  2 (0.5) | 117 (23.7)  16 (3.2)  51 (10.3)  14 (2.8)  13 (2.6)  22 (4.5)  2 (0.4) |
| **Musculoskeletal agents**  NSAIDs  COX-2 inhibitors  Antigout  Anti-osteoporotic agents | 62 (16.8)  41 (11.1)  21 (5.7)  1 (0.3)  4 (1.1) | 67 (13.6)  44 (8.9)  22 (4.5)  1 (0.2)  5 (1.0) |
| **Antithrombic agents**  Antiplatelet agents  Aspirin  Cilostazole  Anticoagulant agents  DOAC  Warfarin | 49 (13.3)  37 (10.1)  19 (5.2)  12 (3.3)  12 (3.3)  7 (1.9)  5 (1.4) | 102 (20.6)  69 (14.0)  36 (7.3)  18 (3.6)  33 (6.7)  17 (3.4)  16 (3.2) |
| **Psychotropic agents**  Benzodiazepines  Antipsychotics  Hypnotics | 39 (10.6)  23 (6.3)  11 (3.0)  4 (1.1) | 48 (9.7)  26 (5.3)  17 (3.4)  4 (0.8) |
| **Antidiabetic agents**  Sulfonylureas  Insulins  DPP-4 inhibitors  Metformin | 37 (10.1)  15 (4.1)  13 (3.5)  3 (0.8)  2 (0.5) | 39 (7.9)  15 (3.0)  14 (2.8)  3 (0.6)  2 (0.4) |
| **Psychoanaleptics** | 18 (4.9) | 18 (3.6) |
| **Antineoplastic agents** | 17 (4.6) | 20 (4.0) |
| **Antimicrobial agents** | 13 (3.5) | 19 (3.8) |
| **Herbal medications** | 12 (3.3) | 17 (3.4) |
| **Antiepileptic agents** | 10 (2.7) | 11 (2.2) |
| **Antihistamines** | 5 (1.4) | 8 (1.6) |
| **Urologicals** | 2 (0.5) | 2 (0.4) |
| **Opioids** | 2 (0.5) | 4 (0.8) |
| **Antiparkinson drugs** | 1 (0.3) | 1 (0.2) |

^a^These categories of medications were based on the World Health Organization Anatomical Therapeutic Chemical classification.

^b^A total of 368 medications resulted in 287 hospitalizations.

ADR, adverse drug reaction; COX-2, cyclooxygenase-2; DOAC, direct-acting oral anticoagulant; DPP-4, dipeptidyl peptidase IV; NSAIDs, nonsteroidal anti-inflammatory drugs; RAS, renin-angiotensin system.

**eTable 4.** Comparison of the prevalence of hospital admissions due to ADRs between the pre-COVID-19 pandemic and COVID-19 pandemic periods.

| **Period** | **Total number of hospitalizations** | **Number of hospitalizations due to ADRs, n (%)** | ***P* value^a^** |
| --- | --- | --- | --- |
| Pre-COVID-19 pandemic period  (from July 2018 to January 2020) | 3049 | 186 (6.1%) | < 0.001 |
| COVID-19 pandemic period  (from February 2020 to May 2021) | 2658 | 101 (3.8%) |  |

^a^The prevalence of hospital admissions due to ADRs between the pre-COVID-19 pandemic and COVID-19 pandemic periods was compared using Fisher’s exact test. The level of significance was set at 5%.

ADR, adverse drug reaction; COVID-19, coronavirus disease 2019.
